# A novel variant in the *FZD4* gene leading to familial exudative vitreoretinopathy: A case report and literature review

**DOI:** 10.1097/MD.0000000000045894

**Published:** 2025-11-07

**Authors:** Bihong Yang, Lixing Zhou, Chunhong Ye, Chunjuan Wang, Xiaofang Lan, Weihao Liu, Penglong Chen, Jiao Liu

**Affiliations:** a Lishui Key Laboratory of Birth Defect Prevention and Control, Lishui Maternity and Child Health Care Hospital, Lishui, China; b Department of Optometry and Ophthalmology College, Wenzhou Medical University, Wenzhou, China.

**Keywords:** case report, familial exudative vitreoretinopathy, *FZD4* gene, genetic etiology, whole-exome sequencing

## Abstract

**Rationale:**

Familial exudative vitreoretinopathy (FEVR) is a genetically heterogeneous retinal vascular disorder. This report describes a novel *frizzled class receptor 4 (FZD4*) gene mutation (c.977C>T) identified in a FEVR pedigree and highlights its phenotypic variability and clinical management.

**Patient concerns:**

A neonatal proband was referred for routine RetCam III fundus screening on postnatal day 3. No systemic abnormalities were reported, but retinal abnormalities were detected.

**Diagnoses:**

Fundus examination revealed asymmetric retinopathy, with a white temporal demarcation line in the right eye and extensive avascular zones with exudative proliferation in the left. Genetic testing confirmed a heterozygous *FZD4* mutation (c.977C>T) in the proband and affected family members, consistent with autosomal dominant FEVR.

**Interventions:**

The proband received prophylactic retinal photocoagulation in both eyes and underwent regular ophthalmic follow-up. Family members with mild retinal changes were monitored.

**Outcomes:**

At 12 months of age, the proband achieved normal visual acuity with stable retinal status. Adult carriers exhibited only mild peripheral vascular anomalies, suggesting incomplete penetrance and variable expressivity.

**Lessons:**

This case expands the mutation spectrum of *FZD4* and underscores the importance of neonatal fundus screening and genetic analysis for early detection of FEVR. Prompt intervention can preserve visual function despite underlying genetic heterogeneity.

## 1. Introduction

Familial exudative vitreoretinopathy (FEVR) is a hereditary ocular disease that can result in blindness and typically affects both eyes. It is primarily characterized by the abnormaldevelopment of retinal vasculature in the peripheral retina, with fundus examinations frequently revealing avascular regions, especially in the temporal periphery.^[1]^ The clinical presentation of FEVR is highly heterogeneous, exhibiting considerable variability among families, individual patients, and even between the eyes of the same individual. In mild cases, symptoms may be absent; however, as the disease progresses, complications such as retinal detachment and blindness may occur. The inheritance patterns of FEVR are diverse, encompassing autosomal recessive, X-linked recessive, and autosomal dominant modes of transmission.^[2,3]^ To date, 11 causative genes have been identified,^[4]^ among which the frizzled class receptor 4 (*FZD4*) gene, located on chromosome 11q14.2, is particularly noteworthy. This gene comprises 2 exons and encodes the *FZD4* protein, consisting of 537 amino acids,^[5]^ and follows an autosomal dominant inheritance pattern.^[6]^ The *FZD4* protein is essential for maintaining normal retinal vascular development and photoreceptor cell function.^[7]^ Structurally, *FZD4* protein includes an N-terminal signal peptide (amino acids 1–36) that facilitates proper membrane insertion, an extracellular cysteine-rich domain (amino acids 40–161) that binds Wnt ligands, a 7-transmembrane domain (amino acids 161–221) forming 3 intracellular and 3 extracellular loops, and a C-terminal domain (amino acids 221–537).^[8]^

This study highlights a unique case of FEVR in a child caused by an *FZD4* gene mutation. The specific symptoms presented in this case offer important insights into the pathogenesis and management of FEVR associated with *FZD4* mutations. This case contributes to the limited knowledge of rare but significant complications in FEVR, underscoring the need for comprehensive clinical evaluation and genetic analysis among family members of hereditary eye diseases for accurate diagnosis and effective treatment.

## 2. Materials and methods

### 2.1. Case presentation

This study focused on a neonate who was delivered via spontaneous vaginal delivery at the Maternal and Child Health Hospital in Lishui City on March 5, 2021. On the third day postpartum, a fundus examination was conducted using the RetCam III wide-field fundus imaging system. Informed consent was obtained from the guardian of the patient, following which we collected the medical history and pertinent examination data. Subsequently, whole-exome sequencing and Sanger sequencing were performed. The study received approval from the Medical Ethics Committee of our institution, and all procedures complied with established ethical guidelines. A patient perspective was not applicable due to the proband’s neonatal status. But the family expressed full adherence to follow-up and treatment recommendations.

### 2.2. Methods

#### 2.2.1. Clinical data collection

The study was approved by the Ethics Committee of Lishui Maternal and Child Health Care Hospital (Approval No.: 202030). Written informed consent was obtained from the proband’s guardian for publication. The critical role of parental consent and their supportive involvement in the long-term management plan was highlighted throughout the process. Comprehensive clinical data were collected, encompassing the patient’s medical history, findings from physical examinations, results from ancillary examinations, laboratory test outcomes, and ophthalmic examination results.

#### 2.2.2. DNA sequencing

Sequencing was conducted using the Illumina NovaSeq platform. Target region capture and library construction were performed with the SureSelect XT Human All Exon V6 kit (Agilent). Paired-end sequencing (2 × 150 bp) was executed, and Sanger sequencing was subsequently employed for the familial validation of the identified variants.

#### 2.2.3. Bioinformatics analysis

The high-throughput sequencing data, represented by Fastq files, underwent processing to exclude low-quality reads. The remaining sequences were subsequently aligned to the human reference genome utilizing the Burrows-Wheeler Aligner method. Mutation sites within the target sequences were identified using the Genome Analysis Toolkit. These mutation sites were annotated using Annovar software, referencing public mutation databases. To predict the potential impact of the mutations on protein function, we evaluated factors such as the mutation’s frequency in the general population, sequence conservation, amino acid alterations, and the mutation’s position within the protein structure. In accordance with the guidelines of the American College of Medical Genetics and Genomics, the pathogenicity of the mutations was interpreted, taking into consideration the clinical phenotype of the patient.

## 3. Results

### 3.1. Medical history

The patient, a neonate, was born at 39 weeks of gestation via spontaneous vaginal delivery at the Maternal and Child Health Hospital in Lishui City on March 5, 2021, with a birth weight of 3250 g. There was no history of birth asphyxia or requirement for oxygen supplementation, and the mother’s pregnancy was uncomplicated. The infant received phototherapy for neonatal hyperbilirubinemia on postnatal day 2, with no ocular complications reported. No prior ocular interventions were documented in family members.

### 3.2. Ophthalmologic examination results

**Figure 1.**
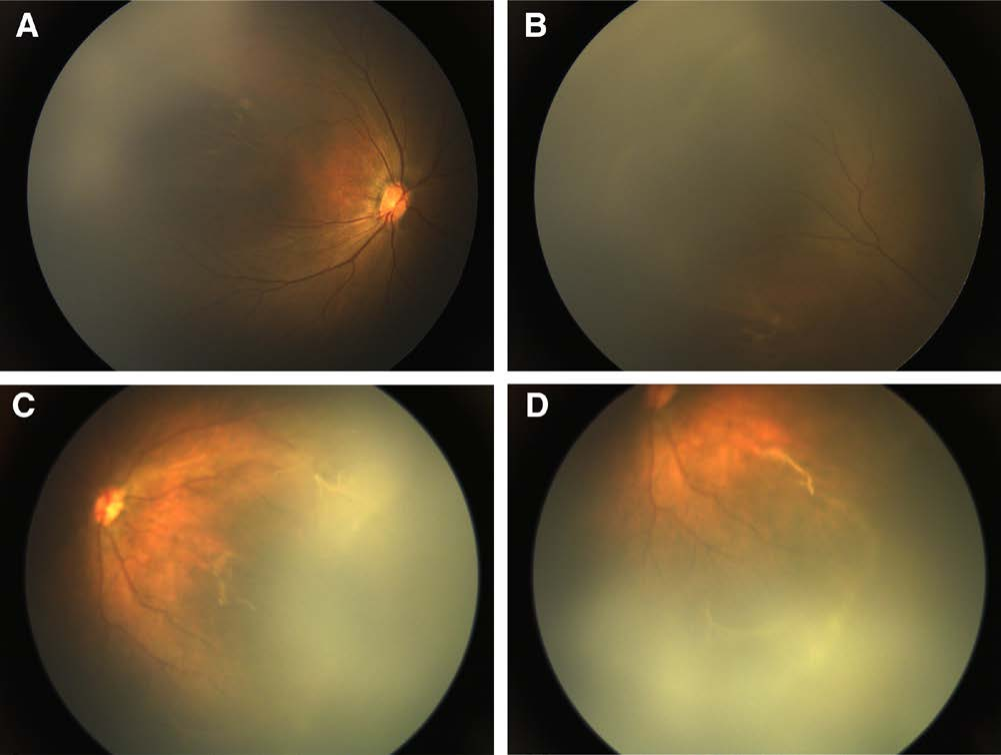
Fundus examination of the proband. (A, B) Right eye: white demarcation line in the temporal periphery. (C, D) Left eye: white demarcation line in zone II, large avascular area in the temporal periphery, and exudation with proliferative membranes in the inferior and temporal regions.

On the third day postpartum, neonatal ocular screening was performed. Examination showed normal ocular morphology and red reflex. RetCam III imaging revealed a white demarcation line in the temporal periphery of the right eye and a similar line in zone 2 of the left eye, along with a significant avascular zone in the left eye’s temporal periphery (Fig. 1A–D). Exudation and proliferative membranes were also noted in the left eye. Based on Pendergast and Trese’s criteria,^[9]^ the patient was provisionally diagnosed with FEVR: stage 1 in the right eye and stage 2B in the left eye.

### 3.3. Systemic and laboratory examination results

Cardiac ultrasound showed a patent foramen ovale (diameter 0.38 cm). Cranial ultrasound revealed mild right lateral ventricle dilation (3.9 mm) and a right choroid plexus cyst (5 mm × 3 mm). Abdominal ultrasound identified bilateral cryptorchidism. TORCH panel (IgG + IgM) showed negative IgM antibodies; Rubella IgG was 55.6 IU/mL, CMV IgG was 52.9 U/mL, HSV-1 IgG was 26.1 COI, with all other markers negative. Irregular blood cell antibody screening was also negative.

### 3.4. Whole exome sequencing and Sanger sequencing results

**Figure 2.**
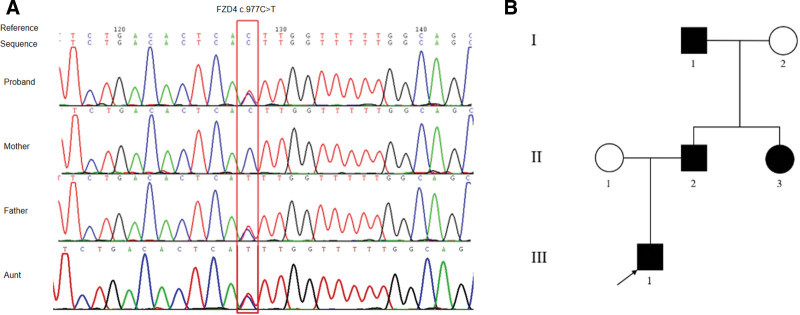
Novel heterozygous FZD4 mutation. (A) Sanger sequencing results of the FZD4 gene in the proband and family members. (B) Pedigree chart. I1: paternal grandfather; I2: paternal grandmother; II1: proband’s mother; II2: proband’s father; II3: proband’s aunt; III1: proband (indicated by arrow).

Whole exome sequencing identified a heterozygous mutation in *FZD4* (NM_012193.4): c.977C>T (p.Thr326Ile) in exon 2, inherited from the father. Sanger sequencing confirmed these findings. The father and aunt were carriers of the c.977G>A mutation in *FZD4* exon 2, while other family members tested negative (Fig. 2A).

### 3.5. Bioinformatics analysis

**Figure 3.**
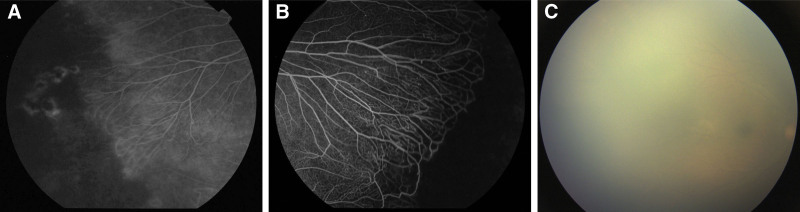
Fundus findings in family members. (A) Father: fluorescein angiography showing an avascular zone with peripheral leakage. (B) Aunt: fluorescein angiography revealing an avascular zone in the peripheral retina. (C) Grandfather: fundus photograph showing an avascular peripheral zone, partially obscured by cataracts.

The *FZD4* mutation c.977C>T (p.Thr326Ile) was not found in Human Gene Mutation Database (HGMD), ClinVar, Genome Aggregation Database, Exome Aggregation Consortium, or 1000 Genomes Project, supporting PM2 criterion. The *FZD4* gene mutation c.977C>T (p.Thr326Ile) leads to an amino acid substitution from threonine to isoleucine at position 326. This particular mutation has not been documented in the HGMD or ClinVar, nor is it present in the Genome Aggregation Database, Exome Aggregation Consortium, 1000 Genomes Project, or local databases, aligning with PM2_Supporting criterion. Among the 148 nonsense and missense variants documented in the HGMD database (2024.04 release) 14 are located within the region between transmembrane domain 4 and 5 (amino acids 324–393). Of these, 13 are missense variants associated with disease causation, suggesting that the region between amino acids 324 and 393 constitutes a mutation hotspot. The variant of this study is within this hotspot (PM1). According to the American College of Medical Genetics and Genomics guidelines, the clinical significance of this mutation is classified as a likely pathogenic variant (LP), based on criteria PM1, PM2_supporting, PP1, PP3, and PP4. The diagnosis was complicated by the phenotypic heterogeneity within this family. While the proband exhibited severe bilateral retinopathy with exudative proliferation, adult carriers (father and aunt) displayed only mild peripheral vascular anomalies (Fig. 3). This variability initially obscured the genetic etiology, delaying definitive diagnosis until whole-exome sequencing identified the pathogenic *FZD4* variant.

### 3.6. Family medical history

Family history was significant for autosomal dominant inheritance of the identified *FZD4* mutation. The proband’s father has had myopia (–3.0 to –4.0 D) since age 20 but maintained normal corrected visual acuity. The mother had unaided visual acuity of 5.0 in both eyes with no remarkable ocular history. Ophthalmic evaluation of relatives revealed peripheral retinal changes and microvascular anomalies (Fig. 3). Specifically, the asymptomatic father (III:3), a confirmed mutation carrier, showed a peripheral avascular zone with subtle vascular leakage on fluorescein angiography (FFA). The affected aunt (II:2) demonstrated a more severe phenotype: FFA identified a larger avascular area with a sharp demarcation line between vascularized and nonperfused retina. Although limited by cataract obscuration, fundus examination of the grandfather (I:1) also indicated peripheral retinal avascularization. The grandmother was unaffected.

This spectrum of clinical presentations—ranging from an asymptomatic carrier to overt disease—confirms the pathogenicity of the *FZD4* variant and illustrates the variable expressivity and incomplete penetrance typical of FEVR. A family pedigree is presented in Figure 2B.

### 3.7. Diagnosis and differential diagnosis

The diagnosis of familial exudative vitreoretinopathy (FEVR) due to an *FZD4* mutation was confirmed by whole-exome sequencing and validated with Sanger sequencing. Differential diagnoses included: Retinopathy of Prematurity, unlikely due to full-term birth and no history of oxygen supplementation; Coats disease, ruled out by bilateral involvement and positive family history; Norrie disease, excluded by absence of systemic manifestations and negative *NDP* mutation; Persistent Fetal Vasculature, ruled out by fundus exam findings. Although retinal findings were observed early, the presence of mild phenotypes in adult carriers and the absence of family history complicated definitive diagnosis. Genetic heterogeneity and variable expressivity further challenged clinical interpretation, underscoring the need for genomic testing to establish diagnostic certainty.

### 3.8. Treatment and follow-up

The patient was referred to a tertiary care center for management. Pattern laser photocoagulation of the avascular retina was performed, followed by 2 monthly intravitreal ranibizumab injections and subsequent pro re nata dosing guided by fluorescein angiography.

During 6 months of follow-up, the patient remained under close surveillance. By 5 months, myopia (−5.0 DS) and astigmatism (−3.0 DC) were detected in the left eye. At 11 months, myopia stabilized, and retinal vasculature advanced to stage III. At 18 months, fluorescein angiography revealed persistent leakage in the left eye, necessitating repeat laser photocoagulation.

At the latest evaluation, best-corrected visual acuity was 5.0 in both eyes. Refractive status was −1.50 DS in the right eye and −7.0 DS/−3.0 DC in the left eye. No treatment-related adverse events (e.g., vitreous hemorrhage, intraocular pressure elevation, or cataract formation) occurred during the 18-month follow-up. Despite current stability, lifelong monitoring is required due to the risk of progressive retinal detachment and refractive error progression. The family maintained full adherence to scheduled visits and treatment protocols.

### 3.9. Guardian’s perspective

The parents described the early detection through neonatal screening as a pivotal “blessing in disguise,” enabling timely intervention and preservation of vision. They expressed deep gratitude toward the medical team for both the initial screening and ongoing care, and reaffirmed their commitment to long-term treatment and follow-up.

## 4. Discussion

Familial exudative vitreoretinopathy (FEVR) represents a genetically heterogeneous retinal disorder characterized by incomplete retinal vascularization and potential sight-threatening complications. To date, 11 pathogenic genes (*NDP, LRP5, TSPAN12, FZD4, KIF11, ZNF408, CTNNB1, JAG1, RCBTB1, ATOH7, ILK4*) have been implicated in its pathogenesis, with most converging on the Wnt and Norrin-β-catenin signaling pathways.^[1,10]^ Emerging evidence suggests a genotype–phenotype correlation, where the number and functional impact of mutations may determine disease severity through pathway-specific disruptions.^[1]^

The *FZD4* gene (11q14.2), located at 11q14.2, the first identified autosomal dominant FEVR-associated gene, encodes a critical Wnt receptor regulating both canonical Wnt/β-catenin and noncanonical Wnt/Ca^2+^ pathways.^[8]^ Its interaction with the Norrin ligand is essential for retinal angiogenesis and blood-retina barrier maintenance.^[11,12]^ Recent structural analyses have mapped pathogenic missense mutations to key functional domains, including extracellular Norrin-binding regions, transmembrane helices (TM1/TM7), and intracellular DVL2 recruitment sites.^[13]^ Notably, *FZD4* dysregulation extends beyond FEVR pathology, with emerging roles in oncogenesis including melanoma and acute myeloid leukemia.^[8]^ This compellingly underscores the necessity of integrating comprehensive clinical evaluation with genetic analysis for accurate FEVR diagnosis and management.

Critically, it highlights the importance of early intervention guided by genetic insights and personalized treatment strategies to improve outcomes. Furthermore, the findings emphasize the vital role of incorporating genetic analysis into neonatal screening programs. Early detection of even subtle retinal changes in asymptomatic carriers is crucial, as it carries significant implications for offspring. Such proactive identification enables timely intervention, potentially preventing severe visual impairment in hereditary retinal disorders, thereby reinforcing the imperative for genetic counseling and family-wide screening.

Clinical progression exhibits marked heterogeneity across age groups. While pediatric cases frequently demonstrate rapid progression to retinal detachment,^[14]^ adult presentations range from asymptomatic carriers to bilateral blindness. Intrafamilial variability in disease severity and asymmetric ocular involvement are common, even among individuals sharing identical mutations.^[15,16]^ This phenotypic diversity may reflect epigenetic modifiers or stochastic developmental factors, as exemplified by the more severe manifestations observed in offspring compared to mutation-positive parents^[15]^—a pattern corroborated in our case series.

The novel *FZD4* c.977C>T (p.Thr326Ile) variant identified in our family pedigree exemplifies this mechanistic link between genotype and phenotype. This mutation resides within the crucial TM5 domain, a recognized mutational hotspot for FEVR. The substitution of polar threonine with hydrophobic isoleucine is predicted to destabilize the α-helical structure and disrupt the local hydrophobic core. This alteration likely impairs critical protein functions, such as membrane integration or conformational changes required for Wnt signal transduction. The consequent failure in activation of the Wnt signaling pathway activation provides a direct mechanistic explanation for the aberrant retinal vascular development observed in our patients, demonstrating a clear genotype–phenotype correlation.

The pathognomonic feature of avascular peripheral retina, best visualized through FFA,^[2]^ creates a permissive environment for neovascular complications. Wide-field imaging systems, particularly Retcam III, as utilized in our diagnostic protocol, now complement FFA by enhancing detection of temporal avascular zones and subclinical lesions.^[17]^ These technological advances address the diagnostic challenges posed by the disease’s variable expressivity and unpredictable progression timelines.

Therapeutic strategies emphasize early intervention to disrupt the ischemic-neovascularization cascade. For stage 1 to 2 disease, laser photocoagulation remains the mainstay treatment, effectively reducing vitreoretinal traction through ablation of avascular retina.^[18]^ While anti-VEGF agents show promise in managing neovascularization,^[19]^ their rapid exudate resolution may paradoxically increase tractional forces—a limitation potentially mitigated by anterior chamber delivery methods currently under investigation.^[20]^ Advanced stages (3–5) require surgical intervention, with scleral buckling and pars plana vitrectomy selection dependent on detachment characteristics and contralateral eye status.^[21]^ Combined approaches integrating perioperative anti-VEGF therapy and intraoperative photocoagulation have shown particular efficacy in complex cases.^[19]^

Future therapeutic horizons focus on molecular pathway modulation. The recent identification of EMC1 as a regulator of Wnt/β-catenin pathway highlights potential targets for gene therapy.^[22]^ Pharmacological *FZD4* agonists like SZN-413, which mimic Norrin function to rescue retinal vascular development in preclinical models,^[23]^ represent particularly promising candidates for translational development. These advances underscore the importance of continued genotype–phenotype correlation studies to guide personalized management strategies.

In conclusion, this case report details the clinical and genetic profile of a rare FEVR presentation, advancing our understanding of *FZD4* mutation-associated disease pathogenesis and management. While the rarity limits generalizability, our findings underscore the necessity of integrating detailed clinical evaluation with genetic analysis for accurate FEVR diagnosis and management. Based on our experience, we recommend that clinical practice for families at risk should prioritize: neonatal retinal screening with wide-field imaging and genetic testing to enable presymptomatic diagnosis, cascade screening of all first-degree relatives to identify asymptomatic carriers, and a multidisciplinary, long-term management strategy guided by genetic insights. This approach of early intervention and personalized care is critical for improving visual outcomes. Furthermore, the early detection of subtle retinal changes, even in asymptomatic carriers, carries significant implications for genetic counseling and family planning. Such proactive identification enables timely intervention, potentially preventing severe visual impairment and thereby reinforcing the imperative for family-wide screening and lifelong monitoring.

## Author contributions

**Data curation:** Chunhong Ye.

**Formal analysis:** Chunhong Ye, Chunjuan Wang, Penglong Chen, Jiao Liu.

**Funding acquisition:** Chunhong Ye, Chunjuan Wang.

**Investigation:** Chunhong Ye, Xiaofang Lan.

**Methodology:** Xiaofang Lan, Jiao Liu.

**Project administration:** Lixing Zhou.

**Resources:** Lixing Zhou, Weihao Liu.

**Software:** Lixing Zhou.

**Supervision:** Weihao Liu.

**Writing – original draft:** Bihong Yang, Lixing Zhou.

**Writing – review & editing:** Bihong Yang, Lixing Zhou, Penglong Chen, Jiao Liu.
